# Comparison of fidaxomicin, thuricin CD, vancomycin and nisin highlights the narrow spectrum nature of thuricin CD

**DOI:** 10.1080/19490976.2024.2342583

**Published:** 2024-05-09

**Authors:** L. Walsh, A. Lavelle, PM O’Connor, C. Hill, R. P. Ross

**Affiliations:** a School of Microbiology, University College Cork, Cork, Ireland; b APC Microbiome Ireland, University College Cork, Cork, Ireland; c Teagasc Food Research Centre, Cork, Ireland

**Keywords:** Gut microbiome, Fidaxomicin, Thuricin CD, Vancomycin, Nisin, *Clostridoides difficile*

## Abstract

Vancomycin and metronidazole are commonly used treatments for *Clostridioides difficile* infection (CDI). However, these antibiotics have been associated with high levels of relapse in patients. Fidaxomicin is a new treatment for CDI that is described as a narrow spectrum antibiotic that is minimally active on the commensal bacteria of the gut microbiome. The aim of this study was to compare the effect of fidaxomicin on the human gut microbiome with a number of narrow (thuricin CD) and broad spectrum (vancomycin and nisin) antimicrobials. The spectrum of activity of each antimicrobial was tested against 47 bacterial strains by well-diffusion assay. Minimum inhibitory concentrations (MICs) were calculated against a select number of these strains. Further, a pooled fecal slurry of 6 donors was prepared and incubated for 24 h with 100 µM of each antimicrobial in a mini-fermentation system together with a no-treatment control. Fidaxomicin, vancomycin, and nisin were active against most gram positive bacteria tested *in vitro*, although fidaxomicin and vancomycin produced larger zones of inhibition compared to nisin. In contrast, the antimicrobial activity of thuricin CD was specific to *C. difficile* and some *Bacillus* spp. The MICs showed similar results. Thuricin CD exhibited low MICs (<3.1 µg/mL) for *C. difficile* and *Bacillus firmus*, whereas fidaxomicin, vancomycin, and nisin demonstrated lower MICs for all other strains tested when compared to thuricin CD. The narrow spectrum of thuricin CD was also observed in the gut model system. We conclude that the spectrum of activity of fidaxomicin is comparable to that of the broad-spectrum antibiotic vancomycin *in vitro* and the broad spectrum bacteriocin nisin in a complex community.

## Introduction

*Clostridioides difficile* is a gram positive, anaerobic, spore-forming bacterium and is the most common cause of hospital-acquired diarrhea.^1^ Recurrence of *C. difficile* infection (CDI) in recovered patients who have undergone antibiotic treatment is common, with a reported rate of relapse estimated at 25% in the United States of America (USA).^2^ These infections continue to be a prominent problem for healthcare providers and patients alike, contributing to higher healthcare costs as well as increased mortality and morbidity.^3^ The ability of *C. difficile* to form spores increases its capacity to persist on environmental surfaces and subsequently aid in its spread.^1^ The 2019 Centre for Disease Control Antibiotic Resistance Threat report concluded that CDI is an urgent threat. An estimated 223,900 people required medical assistance for *C. difficile* infection in the USA − 12,800 of whom died as a result of their infection.^4^ CDI is associated with nosocomial diarrhea, accounting for 15 to 25% of antibiotic-related cases.^5^

*C*. *difficile* is an opportunistic pathogen, known to colonize patients who have undergone antibiotic therapy.^6^ Ordinarily, commensal gut bacteria can provide colonization resistance to many pathogenic bacteria through production of antimicrobials and/or nutrient competition. However, antibiotics can impact the microbiome and permit the growth of pathogenic bacteria, such as *C. difficile*. Many antibiotics have been reported to play this role in facilitating CDI recurrence, including vancomycin, metronidazole, ampicillin, amoxicillin, fluoroquinolones, and cephalosporins.^7^

The epidemiology of *C. difficile* has changed in recent decades as carriage by healthy individuals is becoming more prominent.^8–10^ This, tied with an increase in relapse of patients, as well as the reduced effectiveness of frontline treatments, highlights the threat of *C. difficile*.^11,12^ One superior treatment option is fecal microbiota transplants (FMT) which have been shown to prevent recurrence in 80 to 90% of *C. difficile* infections.^5^ FMTs restore diversity and facilitate the engrafting of beneficial bacteria and bacteriophages into a gut microbiome.^13^

Traditionally, a combination of vancomycin and metronidazole is used for the treatment of severe cases of CDI.^14^ Oral vancomycin is also sometimes prescribed as a prophylactic measure as well as a response to infection.^15^ However, there are limitations to vancomycin treatment. Vancomycin-resistant enterococci (VRE) have been known to colonize the gut following antibiotic treatment. In addition, *C. difficile* has been reported to colonize the gut more efficiently once vancomycin treatment has ceased.^16^ Although these treatments are successful at inhibiting initial infection, recurrence of infection can be high. It is reported that within 3 months 15 to 30% of patients relapse following treatment with vancomycin and metronidazole.^5^

Fidaxomicin is an 18-membered macrolactone produced by *Actinoplanes deccanensis* and *Dactylosporangium aurantiacum*.^17^ This antibiotic was approved for the treatment of CDI in 2011 by the United States Food and Drug Administration (FDA) and the European Medicines Agency (EMA).^18^ Fidaxomicin is considered a narrow-spectrum antibiotic, which has demonstrated similar results to vancomycin in terms of clinical cure of CDI following treatment. Treatment with fidaxomicin has been shown to reduce the likelihood of recurrence with CDI.^6^ Fidaxomicin is active against various gram positive bacteria such as clostridia, enterococci, and staphylococci.^19^ Yet, the antibiotic is reported to have minimal effects on the commensal gut bacteria of patients with CDI.^20^

Non-antibiotic antimicrobials also possess potential to treat CDI. Nisin is a bacteriocin produced by *Lactococcus lactis* that demonstrates antimicrobial activity against most gram positive bacteria.^21^ Since its discovery, nisin has been used primarily as a food preservative, with only recent developments suggesting its potential as a therapeutic.^22^ One characteristic of nisin lending to its desirability for use in the pharmaceutical industry is that it is FDA-approved for use in food.^23^ Nisin has demonstrated antimicrobial activity against various antibiotic-resistant bacteria including *Streptococcus pneumoniae*, enterococci, methicillin-resistant *Staphylococcus aureus* (MRSA) and *C. difficile*.^23^ Thuricin CD is a bacteriocin produced by *Bacillus thuringiensis* DPC 6431. In contrast to nisin, the activity of thuricin CD is specific to *C. difficile* and other gram positive spore-forming bacteria.^7^ Both nisin and thuricin CD have shown activity against *C. difficile (ex vivo)* at levels comparable to that of metronidazole^24,25^

The aim of this study was to compare bacteriocins (nisin and thuricin CD) and antibiotics (fidaxomicin and vancomycin) for their potential use as CDI treatments. Well diffusion assays (WDA) and minimum inhibitory concentrations (MICs) were performed against a range of bacteria that included commensal gut microbes and a selection of pathogens, including *C. difficile,* to determine the spectrum of activity of each antimicrobial compared to the other. A mini-fermentation model was then used to examine the effect of each antimicrobial on *C. difficile* growth and to assess the impact of the antimicrobials on the gut microbiome.

## Materials and methods

### Preparation of antimicrobials

Thuricin CD was purified as described by Rea et al.^7^ Briefly, Brain Heart Infusion (BHI) broth was clarified before autoclaving at 121°C for 15 min by passing through a column containing propan-2-ol – washed XAD beads (Sigma-Aldrich Merck Life Science Limited, Vale Road, Arklow, Co Wicklow, Ireland). *B. thuringiensis* DPC 6431 was subcultured twice in BHI broth at 37°C before use. One liter of BHI broth was inoculated with the culture at 0.1% and incubated shaking at 37°C overnight. The culture was centrifuged at 8,280 × g for 15 min. The cell pellet and supernatant were retained. The cells were resuspended in 200 mL of 70% propan-2-ol (pH 2.0) per 1 L broth and stirred at 4°C for 4 h. The culture supernatant was passed through XAD beads and prewashed with 1 L of distilled water. The column was washed with 500 mL of 30% ethanol, and the inhibitory activity eluted in 400 mL of 70% propan-2-ol (pH 2.0) and was retained (S_1_). The cells that had been resuspended in 70% propan-2-ol (pH 2.0) were centrifuged at 8,280 × g for 15 min, and the supernatant (S_2_) was retained; S_1_ and S_2_ were combined. The propan-2-ol was evaporated using a rotary evaporator (Buchi), and the sample was applied to a 6-g (20 mL) Phenomenex C-18 column preequilibrated with methanol and water. The column was washed with 2 column volumes of 30% ethanol, and the inhibitory activity was eluted in 1.5 column volumes of 70% propan-2-ol (pH 2.0). This preparation was concentrated using rotary evaporation before separation of peptides with HPLC as follows: aliquots of ∼2 mL were applied to a Phenomenex C18 RP-HPLC column (Primesphere 10 μm C18-MC 30; 250 × 10.0 mm; 10 μm) previously equilibrated with 45% acetonitrile and 0.1% trifluoroacetric acid (TFA). The column was subsequently developed in a gradient of 45% acetonitrile containing 0.1% TFA to 65% acetonitrile containing 0.1% TFA from 4 to 40 min at a flow rate of 9.9 mL/min. Biologically active fractions were identified using *C. difficile* as a target organism in well-diffusion assays (WDA). Fractions containing the active peptides were pooled, freeze-dried and frozen at − 20°C until use.

Purified nisin and vancomycin (Sigma-Aldrich) were resuspended in autoclaved milli-Q water and then subsequently filter sterilized. Purified fidaxomicin (MedChemExpress) was resuspended in 70% ethanol and then filter sterilized. Purified thuricin CD (as described above) was resuspended in 70% propan-2-ol. Dilutions of all antimicrobials were carried out using sterile mili-Q water.

### Bacterial strains

*S*. *aureus* Newman, MRSA 5824, *L. lactis* spp. *cremoris* HP, *Bacillus firmus* APC 6349, *C. difficile* DSMZ 1696, *Blautia producta* DSMZ 2950, *Enterococcus faecium* APC 1031, *Ruminococcus gnavus* JCM 659ST, *Bifidobacterium longum* ATCC 15,707 and *Enterococcus faecalis* OG1RF were used for determination of MICs of each antimicrobial. For WDA, the list of target organisms, their sources, and growth conditions are outlined in Table 1.

**Table 1.** List of bacterial strains used and their growth conditions. MRSA – methicillin-resistant *Staphylococcus aureus*, VISA – vancomycin intermediate *Staphylococcus aureus*.

Species	Strain	Growth conditions		
		Media	Temp (°C)	Atmosphere
*Staphylococcus aureus*	DPC 5243	BHI	37	aerobic
*Staphylococcus aureus*	DPC 7073	BHI	37	aerobic
*Staphylococcus aureus*	Newman	BHI	37	aerobic
VISA	32679	BHI	37	aerobic
*Staphylococcus epidermidis*	DPC 5990	BHI	37	aerobic
*Staphylococcus capitis*	APC 2923	BHI	37	aerobic
MRSA	DPC 5684	BHI	37	aerobic
*Staphylococcus intermedius*	DSM 20373	BHI	37	aerobic
*Streptococcus agalactiae*	ATCC 13813	BHI	37	aerobic
*Streptococcus pyogenes*	DPC 6992	BHI	37	aerobic
*Streptococcus uberis*	DPC 5344	BHI	37	aerobic
*Streptococcus salivarius*	APC 138	BHI	37	aerobic
*Streptococcus pneumoniae*	APC 3849	BHI	37	aerobic
*Lactiplantibacillus plantarum*	wcfsI	mMRS	37	aerobic
*Limosilactobacillus fermentum*	APC 2582	mMRS	37	aerobic
*Lactobacillus delbrueckii* subsp. *bulgaricus*	LMG 6901	mMRS	37	anaerobic
*Lactobacillus acidophilus*	DPC 3323	mMRS	37	anaerobic
*Lactobacillus helveticus*	DPC 5308	mMRS	37	aerobic
*Ligilactobacillus salivarius*	APC 2096	mMRS	37	aerobic
*Enterococcus faecium*	DPC 3675	BHI	37	aerobic
*Enterococcus faecium*	DPC 25644	BHI	37	aerobic
*Enterococcus faecium*	APC 1031	BHI	37	aerobic
*Enterococcus faecalis*	5152	BHI	37	aerobic
*Enterococcus faecalis*	OG1RF	BHI	37	aerobic
*Enterococcus durans*	EM138	BHI	37	aerobic
*Listeria monocytogenes*	33007	BHI	37	aerobic
*Listeria monocytogenes*	EDG-e	BHI	37	aerobic
*Listeria monocytogenes*	L028	BHI	37	aerobic
*Listeria innocua*	DPC 3572	BHI	37	aerobic
*Lactococcus lactis*	MG 1614	GM17	30	aerobic
*Lactococcus lactis* subsp. *cremoris*	HP	GM18	30	aerobic
*Lactococcus lactis*	MG 1363	GM19	30	aerobic
*Bacillus firmus*	APC 6349	BHI	37	aerobic
*Bacillus thuringiensis*	DPC 6341	BHI	37	aerobic
*Bacillus subtilis*	S249	BHI	37	aerobic
*Bacillus cereus*	DPC 6087	BHI	37	aerobic
*Micrococcus luteus*	DSMZ 1790	BHI	37	aerobic
*Actinomyces oris*	CCUG 34286	BHI	37	anaerobic
*Actinomyces ruminicola*	DPC 7276	BHI	37	anaerobic
*Ruminococcus gnavus*	JCM659ST	YCFA	37	anaerobic
*Ruminococcus gauvreauii*	DSMZ 19829	YCFA	37	anaerobic
*Blautia producta*	DSMZ 2950	YCFA	37	anaerobic
*Bifidobacterium longum*	ATCC 15707	YCFA	37	anaerobic
*Clostridioides difficile*	APC 43	RCM	37	anaerobic
*Clostridioides difficile*	APC 1412	RCM	37	anaerobic
*Clostridioides difficile*	28196	RCM	37	anaerobic
*Clostridioides difficile*	1696	RCM	37	anaerobic

### WDA

The activities of purified nisin, thuricin CD, vancomycin, and fidaxomicin were determined against 47 target organisms using WDA as previously described by Rea et al.^7^ Briefly, cultures were grown overnight in specific broth media and temperatures as specified in Table 1. Twenty mL of the appropriate agar was inoculated with 50 µl of target organism. Once the plate had solidified a well was punched into the agar using a sterilized glass pipette, and 50 µl of each antimicrobial was added into its own well. Plates were incubated under conditions specific to the seeded organism (as described in Table 1). Zones of inhibition (mm) were measured. Relative sensitivity was carried out by measuring the radius of the zone.

### MIC

MIC determinations for strains were performed in triplicate in 96-well microtiter plates (Sarstedt, Sarstedtstraße 1 51588 Nümbrecht Germany) as described previously by van Kraaij et al.^26^ Briefly, to each well of the microtiter plate, 200 μl of phosphate-buffered saline (PBS) containing 1% (wt/vol) bovine serum albumin (BSA) was added, and plates were incubated at 37°C for 30 min. The wells were washed with 200 μl PBS and allowed to dry. The target strains *S. aureus* Newman, *S. aureus* DPC 5824, *L. lactis* subsp. *cremoris* HP, *B. firmus, B. longum* ATCC 15,707, *R. gnavus* JCM659ST, *B. producta* DSMZ 2950, and *E. faecium* APC 1031 were grown overnight under appropriate conditions (Outlined in Table S1). Strains were subcultured into fresh broth, allowed to grow to an OD_600_ of ∼0.5, and diluted to a final concentration of 10^5^ colony forming units (CFUs) ml^−1^ in a volume of 0.2 mL. Nisin, vancomycin, thuricin CD, and fidaxomicin were adjusted to a starting concentration of 200 µg/mL, and 2-fold serial dilutions of each antimicrobial were added to the target strain to final concentrations of 100, 50, 25, 12.5, 6.25, 3.1, and 1.56 µg/mL. The MIC was determined as the lowest concentration that inhibited the growth of the target strain.

### Donor recruitment for faecal fermentation studies

The Clinical Research Ethics Committee of the Cork Teaching Hospitals approved donor recruitment and enrollment (protocol no: APC056). All participants (*n* = 6) were healthy male and female humans aged between 23 and 65 years, who had not consumed antibiotics in the previous 3 months leading up to the study. All donors acknowledged their participation in the study by signing a consent form.

### Fecal medium and model colon

The environment of the distal colon was simulated by the use of fermentation in Hungate tubes. In brief, each antimicrobial (fidaxomicin, thuricin CD, nisin and vancomycin) was tested in triplicate tubes, with three tubes allocated to a no-treatment control. The fecal slurry was prepared under anaerobic conditions by taking 1 g samples of 6 fecal samples and pooling them with the addition of 50 mM phosphate buffer (pH 7.0) containing 0.05% cysteine (Sigma Aldrich, Ireland) to make a 20% slurry w/v. Fecal medium was prepared according to Fooks & Gibson.^27^ The medium was transferred to an anaerobic chamber, where 8 mL was added to each Hungate tube. The final volume of each tube was then made to 10 ml by the addition of 2 mL of 20% fecal slurry. Each tube was then treated with the appropriate antimicrobial at a concentration of 100 µM. The tubes allocated to the no-treatment control contained only fecal medium and fecal slurry. *C. difficile* APC 43 was spiked into each tube to a final concentration of 10^5^ CFU/mL. Hungate tubes were then sealed to maintain anaerobic conditions while the tubes were transferred and incubated in a 37°C shaking incubator (250 rpm) for 24 h.

At time T0, a sample containing 20% fecal slurry and fecal medium at a ratio of 1:5 was taken as the baseline sample. This sample was then frozen at −80°C for DNA extraction. Following incubation for 24 h, 1 mL samples were taken from each tube for DNA extraction. Samples taken at T0 directly after *C. difficile* APC 43 spike-in, as well as samples from each tube after 24 h were serially diluted and plated on CCEY agar (Lab M) containing 1% lysed horse blood (E & O Laboratories Ltd, Burnhouse, Bonnybridge FK4 2HH, United Kingdom). The plates were incubated anaerobically at 37°C for 24 h to detect viable *C. difficile* cells.

### Fecal fermentate bacteriocin extraction treatment

One mL sample of fermentate from each antimicrobial sample was centrifuged at 13,000 rpm for 5 minutes. The supernatant was decanted and filter sterilized into a sterile Eppendorf tube and the remaining cell pellet was resuspended in 150 µL of 70% propan-2-ol (IPA) and 0.1% trifluoroacetic acid (TFA). Tubes were then incubated shaking at room temperature for 4 h. Following this, tubes were centrifuged at 13,000 rpm for 5 min, the supernatant was removed and filter-sterilized into a sterile Eppendorf tube. Samples were then examined for antimicrobial activity against *C. difficile* APC 43 by WDA as described above.

### DNA extraction

One mL samples were taken at T0 and T24 for each condition and frozen at −80°C. DNA was extracted from the samples using the QIAamp® fast DNA stool mini kit (Qiagen 19,300 Germantown Rd Germantown MD 20,874 USA). There were 18 samples, one T0 sample performed in triplicate and five T24 samples (fidaxomicin, thuricin CD, nisin, vancomycin, and no treatment) performed in triplicate. Extractions were carried out according to the Qiagen QIAamp® fast DNA stool mini kit manual.

### Library preparation

Libraries for whole metagenomic sequencing were prepared using the Illumina DNA prep kit (Illumina, San Diego, California, United States) in accordance with the Illumina DNA prep reference guide. Whole metagenomic sequencing was then carried out by Genewiz (Bahnhofstr. 86, 04158 Leipzig, Germany). The data produced from sequencing was then bioinformatically analyzed to determine the change in microbial diversity inferred by each antimicrobial.

### Bioinformatics and statistical analysis

The *C. difficile* colony numbers for T 24 antimicrobial treated and the T24 no treatment control group were compared using paired T-test in GraphPad Prism version 10.1.2. Differences were considered to be statistically significant if *p* ≤ 0.001 (***), *p* ≤ 0.01 (**), *p* ≤ 0.05 (*) or not statistically significant if *p* > 0.05. Raw metagenomic reads underwent quality control assessment using FastQC (https://www.bioinformatics.babraham.ac.uk/projects/fastqc/) and MultiQC (https://academic.oup.com/bioinformatics/article/32/19/3047/2196507). Samples were processed using Kneaddata (https://huttenhower.sph.harvard.edu/kneaddata/) to remove human contamination using the hg37v0.1 database and low-quality reads using default settings. Cleaned metagenomic reads were then processed using the MetaPhlAn4 pipeline^28^ against the chocophlan database (12/2022). All analyses were performed in the R programming language using Rstudio.^29^ Alpha diversity was calculated using the Shannon diversity index. Beta diversity was calculated using the Bray-Curtis divergence on proportion-normalized data. The significance of grouping variables explaining variability in the microbiome data were tested using permutational multivariate analysis of variance (PERMANOVA), with 999 simulations. Ecological analysis was performed using the *vegan* package (version 2.6.2) https://cran.r-project.org/web/packages/vegan/vegan.pdf). Taxonomy barplots present the top 40 most abundant taxa. The plotting library *ggplot2* (version 3.4.1) was used for visualization.^30^

## Results

All antimicrobials tested (bacteriocins nisin and thuricin CD and antibiotics fidaxomicin and vancomycin) are known to act against *C. difficile*. However, their effects on other bacteria inhabiting the gut, such as commensal bacteria, would be expected to vary considerably. We wished to conduct an extensive comparison of these antimicrobials and the collateral damage they cause to the gut microbiome. We directly compared the activity of each antimicrobial on a complex microbial community in a mini fecal fermentation model. WDAs and MICs were performed to determine, the *in vitro* spectrum of inhibition of each antimicrobial.

### Antimicrobial activity in vitro

WDAs were performed using concentrations of 0.1, 1, 10 and 100 μg/mL of each antimicrobial against 47 bacterial isolates. This list of strains was designed to include *C. difficile* as the target organism in CDI and other commensal bacteria, which regularly inhabit the gut. In addition to that, a wide range of other bacterial strains from varying environments have also been selected to examine the full extent of the spectrum of activity of each antimicrobial. WDA were chosen to determine antimicrobial activity as this type of assay is a well established and commonly used assay in bacteriocin studies.^31^ Concentrations of 0.1 and 1 μg/mL of all antimicrobials did not inhibit growth of any strain. The following comparisons are based on a concentration of 10 μg/mL of antimicrobial (Table 2). All antimicrobials produced zones against all four *C. difficile* strains tested. Fidaxomicin exhibited the strongest anti-*C. difficile* activity with an average zone radius of 6.8 mm. Thuricin CD also showed impressive activity against *C. difficile* with an average zone radius of 4.63 mm. Vancomycin and nisin produced average zone radii of 1.68 mm and 0.92 mm, respectively. Fidaxomicin, vancomycin, and nisin were active against most gram positive bacteria tested, although fidaxomicin and vancomycin did produce larger zones of inhibition compared to nisin. In terms of activity against commensal bacteria, fidaxomicin produced large zones of inhibition against *B. longum* ATCC 15,707 (15 mm), *R. gnavus* JCM659ST (6 mm), *B. producta* DSMZ Z950 (9.5 mm), and *Lb. salivarius* APC 2096 (1.5 mm). Nisin and vancomycin were also active against gut commensal bacteria. In contrast, thuricin CD was specifically active against *C. difficile*, *B. cereus* 6087 (1.5 mm), *Listeria innocua* DPC 3572 (1.67 mm) and *B. firmus* APC 6349 (4 mm).

**Table 2.** Activity of fidaxomicin (F), thuricin CD (T), vancomycin (V), and nisin (N) at a concentration of 10 μg/mL against a variety of bacterial strains measured by WDA. The zone size measured in mm was used to determine the color depicted in the Table. Values are means (*n* = 3). APC – APC culture collection, ATCC – American Type Culture Collection, DSMZ- German Collection of Microorganisms and Cell Cultures, DPC – Teagasc Culture Collection, LMG = Laboratorium voor Microbiologie, Universteit Gent, Belgium, JCM – Japan Collection of Microorganisms.

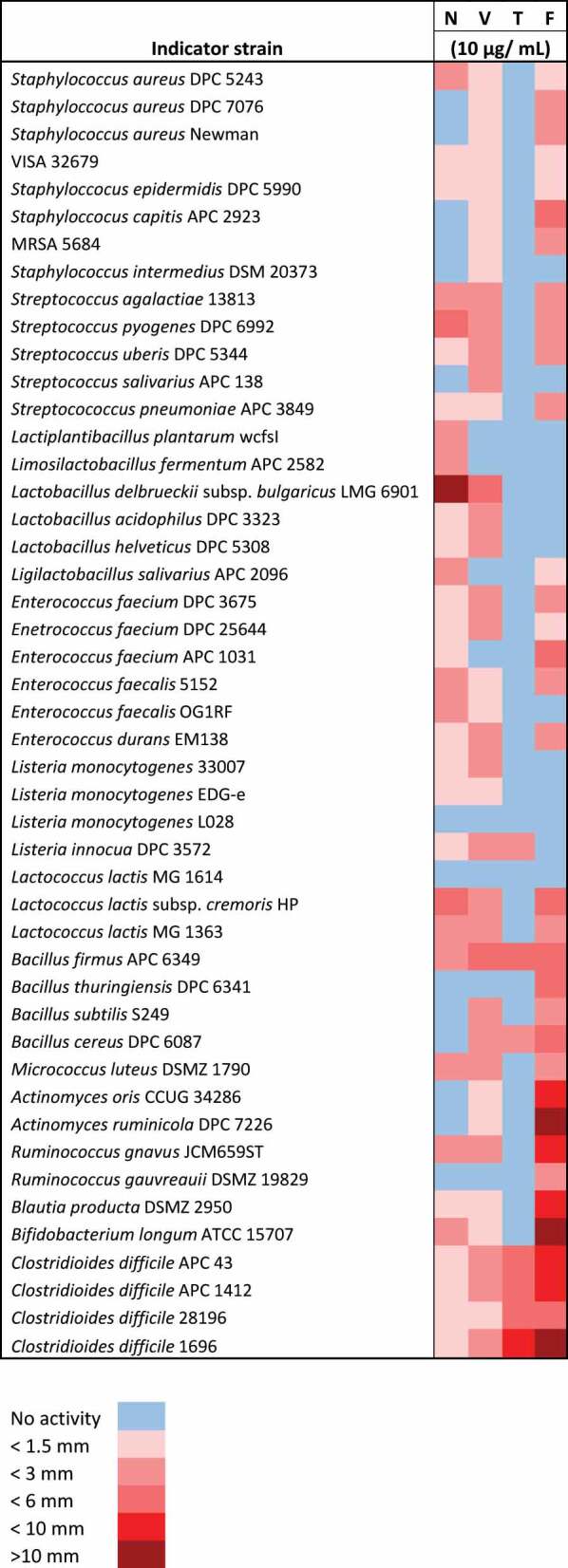	

The MIC analysis reflected the results of the WDAs (Table 3). Thuricin CD demonstrated high levels of activity toward *C. difficile* DSMZ 1696 (<1.56 µg/mL) and *B. firmus* (<3.1 µg/mL). Thuricin CD was also active against *L. lactis* HP (<100 µg/mL), *R. gnavus* JCM659ST (<25 µg/mL) and *B. longum* ATCC 15707 (<50 µg/mL), but only at very high concentrations. Fidaxomicin, vancomycin, and nisin demonstrated lower MICs for all strains tested when compared to thuricin CD with one exception (*C. difficile* DSMZ 1696). All antimicrobials were examined for their MIC against *C. difficile* DSMZ 1696. Fidaxomicin, thuricin CD, and vancomycin had MICs of <1.56 µg/mL, whereas nisin had an MIC of <12.5 µg/mL. When compared on a molar level, nisin has a significantly higher MIC for *C. difficile* DSMZ 1696 (<4.19 mM) than fidaxomicin (<0.16 mM), thuricin CD (<0.44 mM) and vancomycin (<0.23 mM). It is worth noting the MIC of fidaxomicin against *C. difficile* has been reported in the literature to be ≤ 0.001–1 μg/mL.^19^ Similarly for vancomycin and thuricin CD MIC have been reported as 0.464–1.856 µg/mL and 0.703–2.812 µg/mL.^32^ Whereas for nisin Z the MIC of 6.2 μg/mL has been reported in the literature.^33^

**Table 3.** MIC of fidaxomicin, thuricin CD, vancomycin, and nisin against a variety of bacterial strains. Values are means (*n* = 3).

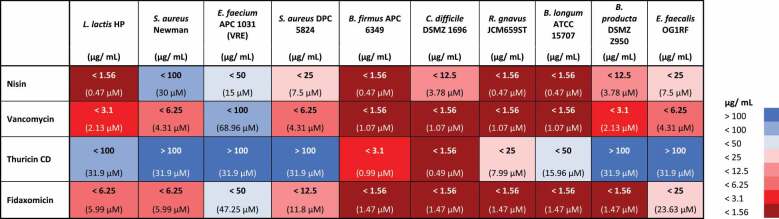	

### Antimicrobial activity in a distal colon model

**Figure 1.**
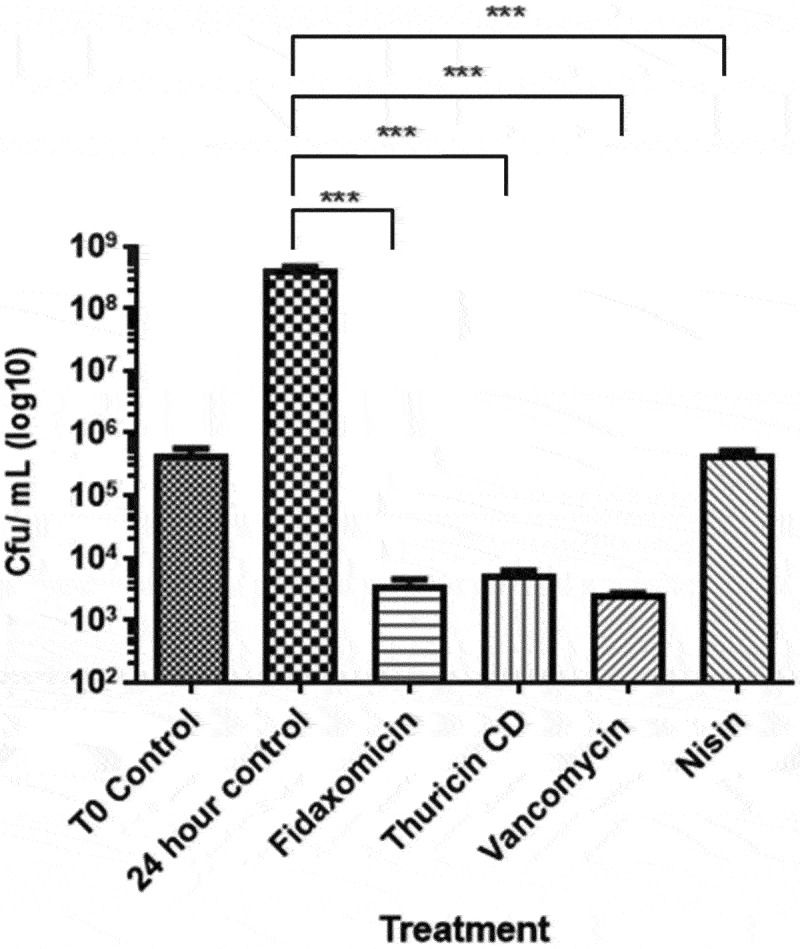
Quantification of *C.* *difficile* cells prior to fermentation and 24 h after incubation for each antimicrobial and the no treatment control. Values are means ± standard error of the mean (SEM) (*n* = 9). ***, *p* ≤ 0.001; **, *p* ≤ 0.01; *, *p* ≤ 0.05.

As complex communities have been shown to require higher treatment concentrations to inhibit growth compared to MIC results, the treatment concentration of each antimicrobial of 100 µM was chosen to ensure *C. difficile* inhibition in the distal colon model.^34^ This concentration has been previously utilized in a distal colon model to examine the effect of nisin on the gut microbiome, resulting in no viable *C. difficile* cells.^25^ The fermentation was carried out for 24 h as this was sufficient time to compare the antimicrobial activity of each antimicrobial against *C. difficile* and other commensal gut bacteria. As this was a batch fermentation a prolonged fermentation would lead to a lack of available nutrients from the media and a buildup of waste products. *C. difficile* APC 43 was spiked into the Hungate tubes to compare the anti-*C. difficile* activity of each antimicrobial following 24 h of fermentation (Figure 1). No *C. difficile* was detected at T0 in the control fecal slurry (data not shown). The post-spike-in fecal slurry contained 10^5^ CFU/mL. The T24 no-treatment control contained 10^8^ CFU/mL, indicating a 1000-fold increase in *C. difficile* levels over the 24-h period. All the antimicrobials resulted in statistically significant reductions in *C. difficile* counts (*p* < 0.001) when compared with the no treatment control after 24 h. Treatment with nisin restricted the level of *C. difficile* to 10^5^ CFU/mL, similar to that found in the T0 samples following spike in. Thuricin CD, vancomycin, and fidaxomicin reduced the levels of *C. difficile* by 4.5 logs when compared to the T24 control levels.

**Figure 2.**
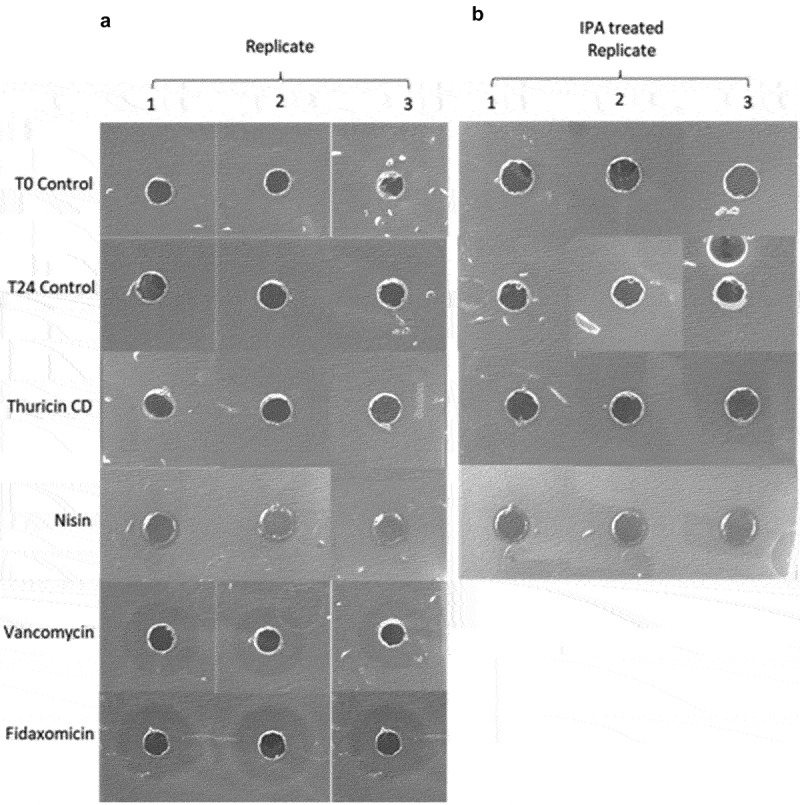
(a) Antimicrobial activity of the fermentate of each sample against *C. difficile* APC 43 after 24 h of fermentation. (b) Antimicrobial activity of fermentate of nisin, thuricin CD, T0 control and T24 control after 24 h of fermentation and treatment with IPA-TFA.

After 24 h the fermentate from each sample was analyzed for antimicrobial activity against *C. difficile* APC 43 by WDA. Fidaxomicin and vancomycin were still active, producing zones of 7 mm and 5 mm, respectively, in comparison to nisin which exhibited only small zones of inhibition (1 mm) and thuricin CD which did not exhibit any zones (Figure 2). For the bacteriocins, IPA-TFA was used to re-solubilize cell-bound peptides into solution. Following IPA-TFA treatment, the zone size of nisin did not change and thuricin CD still did not elicit an antimicrobial effect against the *C. difficile* strain.

**Figure 3.**
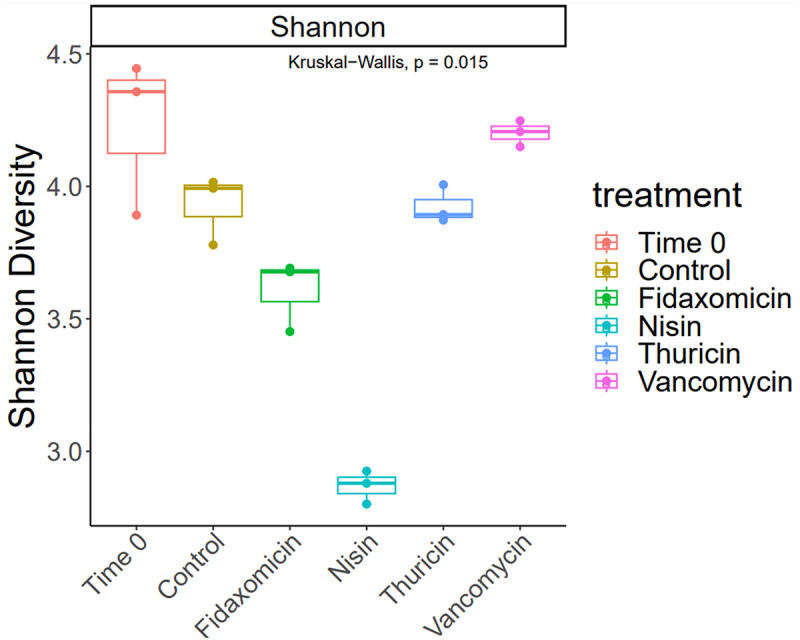
Alpha Diversity presented as Shannon Indices after 24 h across treatment groups. T0 baseline control, No treatment control at 24 h, Fidaxomicin 100 µM at 24 h, Nisin 100 µM at 24 h, Thuricin CD 100 µM at 24 h and vancomycin 100 µM at 24 h.

**Figure 4.**
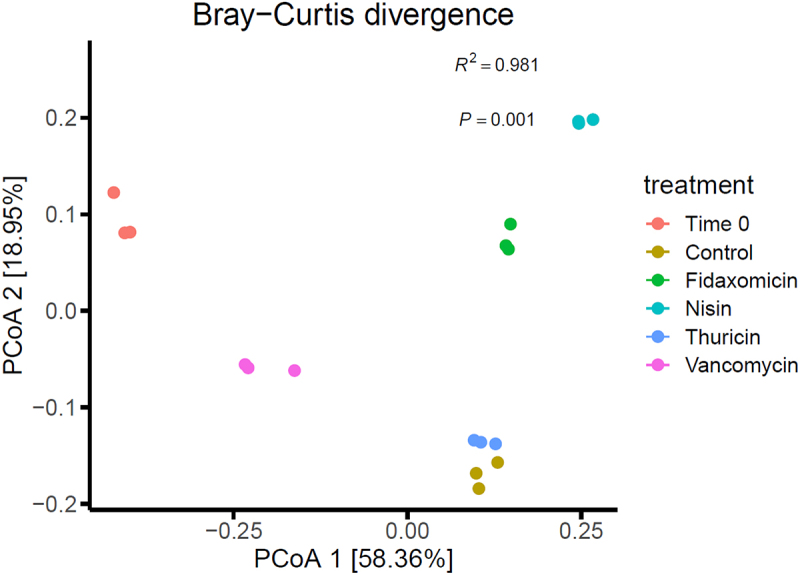
Beta Diversity presented as Bray-Curtis divergence after 24 h across treatment groups. T0 baseline control, No treatment control at 24 h, Fidaxomicin 100 µMat 24 h, Nisin 100 µM at 24 h, Thuricin CD 100 µM at 24 h and Vancomycin 100 µM at 24 h.

**Figure 5.**
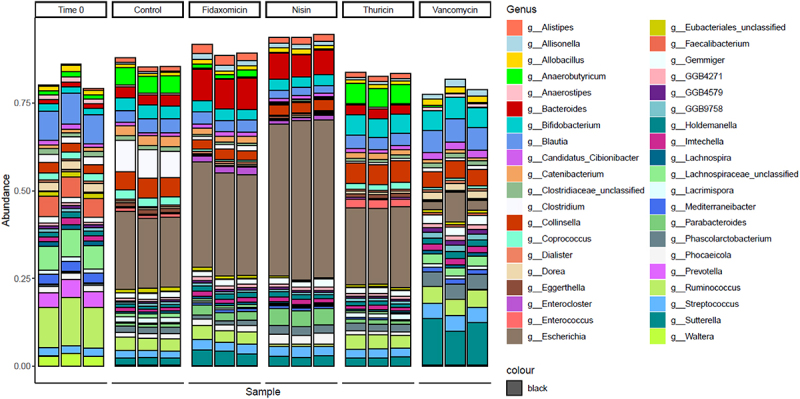
Relative abundance at the genus level across treatment groups.

**Figure 6.**
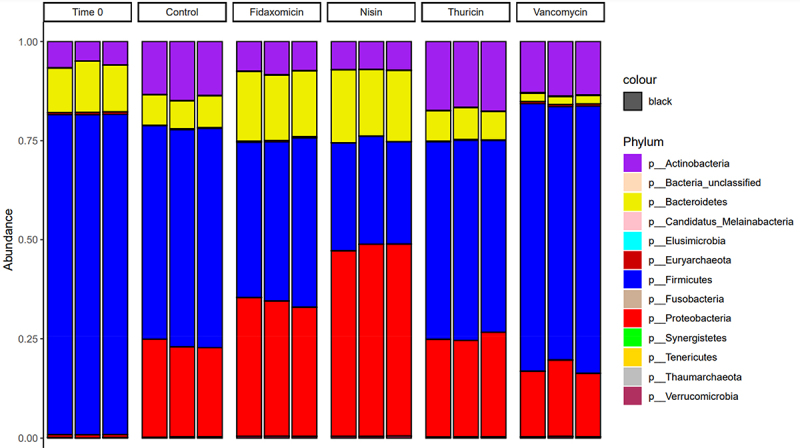
Relative abundance at the phylum level across treatment groups.

Alpha diversity was established using the Shannon index (Figure 3), which showed a similar diversity between the T24 no-treatment control and thuricin CD, with a reduction in the fidaxomicin samples and a further reduction in samples from the nisin group. In contrast, vancomycin appeared to be closer to the T0 control in terms of overall diversity levels. Beta diversity (Figure 4), established using Bray-Curtis divergence, reported a high degree of similarity between the T24 control and thuricin CD samples, compared to that of the fidaxomicin and nisin groups. This indicates that fidaxomicin did not disturb the microbiome to as severe a degree as nisin or vancomycin, but it was not as narrow spectrum as thuricin CD. In terms of relative abundance, *Clostridium* spp. was greatly reduced in all treatment samples when compared to the T24 control, with the most significant reductions observed for nisin, closely followed by thuricin CD (Figure 5). Commensal bacteria such as *Anaerobutyricum* spp., *Anaerostipes* spp., *Coprococcus* spp. and *Enterococcus* spp. were reduced in all treatment groups except for thuricin CD. *Ruminoccus* spp. and *Blautia* spp. were reduced in the nisin group but remained unchanged in all other treatment groups. Other commensal bacteria such as *Streptococcus* spp., *Prevotella* spp. and *Dialister* spp. were unchanged by any treatment group when compared to the T24 no-treatment control. *Bacteroides* spp. and *Escherichia* spp were increased in the fidaxomicin and nisin groups. This is thought to be due to the broad spectrum antimicrobial activity against gram positive bacteria. *Bifidobacterium* spp. were marginally reduced in the fidaxomicin and nisin groups, in contrast, this genus was slightly increased in the thuricin CD and vancomycin groups. A reduction was observed in the diversity of the T24 no-treatment control when compared to the T0 control. This is a relatively normal occurrence observed in studies of a similar design utilizing distal colon fermentation models.^35,36^ At the phylum level, the T0 control was made up by a majority of firmicutes (Figure 6). A large degree of firmicutes were present in all 24 samples as well, however Proteobacteria was observed at a far higher level compared to the T0 control. Similarly to trends noted at the genus level, the T24 nisin and fidaxomicin sample show similar phylum patterns. Whereas, the T24 control and the T24 thuricin treated samples are similar in terms of phylum diversity.

## Discussion

In this study, the bacteriocins nisin and thuricin CD and the antibiotics fidaxomicin and vancomycin were assessed for their efficacies as treatments for *C. difficile*. The ideal treatment must eradicate CDI while also minimally affecting commensal gut bacteria, and so these features were examined for each antimicrobial.

Numerous reports have demonstrated the anti-*C. difficile* activities of all four antimicrobials examined in this study.^6,7,14,23^ The existing data was supported by the results of the MICs generated in this study, in which all antimicrobials were active against *C. difficile* DSMZ 1696. Fidaxomicin, thuricin CD and vancomycin have a lower MIC (<1.56 µg/mL) when compared with nisin (<12.5 µg/mL), indicating nisin is not as active against *C. difficile* as the other antimicrobials. Taking both the MICs and WDAs into account, fidaxomicin was the most impressive at eradicating *C. difficile*. This was followed by thuricin CD, which was also impressively active against all four *C. difficile* strains. Vancomycin was the third most active antimicrobial tested against *C. difficile*. Examination of the anti-*C. difficile* activity in a complex community reflective of the gut microbiome showed that all antimicrobials were effective at reducing *C. difficile* levels. Thuricin CD was the most impressive in terms of consistently exhibiting antimicrobial activity against *C. difficile* supported by the plate culturing results and metagenomic sequencing data.

Vancomycin has long been used as the gold standard treatment for CDI.^14^ However, this treatment has been heavily associated with relapse in patients.^37^ This is thought to be due to the broad-spectrum activity of vancomycin. While it is effective in killing *C. difficile*, other commensal gut bacteria are simultaneously targeted. As expected, vancomycin exhibited a broad spectrum of activity against most of the gram positive strains tested *in vitro*, however, the metagenomic data revealed that the vancomycin-treated samples were capable of maintaining a high degree of diversity albeit very different in composition from the control sample. We suggest that the vancomycin treatment lowered the absolute bacterial numbers and the remaining survivors exhibit a high bacterial diversity. There are no studies to our knowledge reporting maintenance of a high level of diversity in the gut microbiome following vancomycin treatment, with many studies reporting the opposite effect.^38–40^ However, there is also the possibility that the “eagle effect” of vancomycin may have contributed to the ability of a diverse range of bacteria to grow as the treatment concentration used was far above that of the MIC of vancomycin against *C. difficile*, as this phenomenon has been observed in other studies examining the effect of vancomycin on *C. difficile*.^41^

Fidaxomicin is described as a narrow-spectrum antibiotic with minimal effects on the commensal bacteria of the gut.^20,42^ The mechanism of action of fidaxomicin is to inhibit RNA polymerase by means of binding to a residue in the zinc-binding domain of the RNA polymerase β’ subunit. This is commonly found in Clostridia as well as Firmicutes, and gram positive bacteria to a lesser extent.^43^ Impressive anti-*C. difficile* activity supports the prevailing concept that fidaxomicin is the most suitable CDI treatment. However, we have shown that fidaxomicin is active against numerous commensal gut bacteria. In this respect, the spectrum of activity of fidaxomicin is more comparable to that of the broad-spectrum bacteriocin nisin. Thuricin CD is highly specific in its activity, acting only on *C. difficile* and some *Bacillus* and *Listeria* species with minimal effects on gut commensals. This suggests that thuricin CD is superior to fidaxomicin for CDI, in terms of minimizing collateral damage.

One advantage of fidaxomicin is the lack of isolated resistant strains to the antimicrobial. To our knowledge, there have only been five clinically isolated fidaxomicin resistant *C. difficile* strains to date.^44^ This could be because fidaxomicin is a last resort treatment for CDI, with vancomycin generally used as the primary option for treatment, resulting in minimal exposure of fidaxomicin to the environment due to the scarcity of its use. Subsequently leading to less opportunity for the development of bacterial resistance to fidaxomicin. Fidaxomicin and its metabolite OP-1118 have been reported to have a prolonged post-antibiotic effect.^45^ Indeed, as observed in our study, the fermentate treated with fidaxomicin and vancomycin appeared to retain antimicrobial activity at T24, as well as nisin to a lesser degree. In contrast, following 24 h of fermentation, thuricin CD was no longer detectable in the sample. This indicates that fidaxomicin, vancomycin, and nisin remain active against the bacteria in the gut up to 24-h post-treatment. In contrast, thuricin CD is no longer active 24-h post-treatment. One factor in the development of bacterial resistance is prolonged exposure to the antimicrobial, therefore the faster degradation of thuricin CD could be seen as a favorable attribute.

Ridinilazole is a recently identified potential therapeutic for CDI that is reported to have a narrow spectrum of activity. The activity of this antimicrobial has been demonstrated against *C. difficile in vitro*, *in vivo* and in gut models,^46,47^ with *in vitro* inhibitory concentrations similar to that of fidaxomicin (0.015–0.5 µg/mL).^48^ In contrast to fidaxomicin, ridinilazole is not active against a large population of gram positive bacteria, including *S. aureus*, *E. faecalis, E. faecium* and streptococci, and with low activity observed against *Eggerthella lenta, Bifidobacterium* spp, *Peptostreptococcus anaerobius* and *Finegoldia magna*.^49^ Similarly, chlorotonil A (ChA) and its derivative CHB1-Ep2 have also recently been identified as anti-*C. difficile* agents.^50^ ChA is a natural product isolated from myxobacterium *Sorangium cellulosum*. The antimicrobial has the ability to prevent CDI manifestation in mice, and more significantly results in a lower rate of relapse when compared to vancomycin. However, the spectrum of activity of ChA is broad against gram positive bacteria and some fungi^51,52^ This suggests that this antimicrobial may be comparable to that of vancomycin, nisin, and fidaxomicin in terms of collateral damage to the gut microbiome. It would be an interesting comparison to examine the spectrum of activity of ChA and ridinilazole against fidaxomicin and thuricin CD in a complex community model.

We conclude that the spectrum of activity of fidaxomicin is comparable to that of the broad-spectrum antibiotic vancomycin *in vitro* and the broad-spectrum bacteriocin nisin in a gut model. While highly active against *C. difficile*, fidaxomicin does show activity against gut commensal bacteria such as *Bifidobacterium* spp., *Ruminococcus* spp. and some *Lactobacillus* spp. *in vitro*, and *Anaerobutyricum* spp., *Anaerostipes* spp., *Coprococcus* spp., and *Enterococcus* spp. in a gut model. Thuricin CD also demonstrated high activity against *C. difficile*, however in contrast to fidaxomicin, this activity is highly specific and allows gut commensal bacteria to remain relatively unchanged. This is the first time to our knowledge that a mini-fermentation model has been used to compare the effect of fidaxomicin, thuricin CD, vancomycin, and nisin on the gut microbiome.

## Data Availability

The authors declare that all the data supporting our findings in the study are available within the paper.

## References

[1] Johnson S. *A successful collaboration between clinical nurse leaders and clinical nurse specialists combating hospital-acquired*; 2018. http://journals.lww.com/nursingmanagement

[2] Bao H, Lighter J, Dubrovskaya Y, Merchan C, Siegfried J, Papadopoulos J, Jen S-P. Oral vancomycin as secondary prophylaxis for Clostridioides difficile infection. Pediatrics. 2021;148(2). doi:10.1542/peds.2020-031807. 34330867

[3] Shah DN, Aitken SL, Barragan LF, Bozorgui S, Goddu S, Navarro ME, Xie Y, DuPont HL, Garey KW. Economic burden of primary compared with recurrent Clostridium difficile infection in hospitalized patients: a prospective cohort study. J Hosp Infect. 2016;93(3):286–16. doi:10.1016/j.jhin.2016.04.004. 27209056

[4] Centre for disease control and prevention. Antibiotic resistance threats in the United States, 2019. US Department of Health and Human Services, Centres for Disease Control and Prevention: US Department of Health and Human Services, Centres for Disease Control and Prevention; 2019.

[5] Antharam VC, Li EC, Ishmael A, Sharma A, Mai V, Rand KH, Wang GP. Intestinal dysbiosis and depletion of butyrogenic bacteria in Clostridium difficile infection and nosocomial diarrhea. J Clin Microbiol. 2013;51(9):2884–2892. doi:10.1128/JCM.00845-13. 23804381 PMC3754663

[6] Louie TJ, Miller MA, Mullane KM, Weiss K, Lentnek A, Golan Y, Gorbach S, Sears P, Shue Y-K. Fidaxomicin versus vancomycin for Clostridium difficile infection. N Engl J Med. 2011;364(5):422–431. doi:10.1056/NEJMoa0910812. 21288078

[7] Rea MC, Sit CS, Clayton E, O’Connor PM, Whittal RM, Zheng J, Vederas JC, Ross RP, Hill C. Thuricin CD, a posttranslationally modified bacteriocin with a narrow spectrum of activity against Clostridium difficile. Proc Natl Acad Sci. 2010;107(20):9352–9357. doi:10.1073/pnas.0913554107. 20435915 PMC2889069

[8] Al-Zahrani IA. Clostridioides (Clostridium) difficile: a silent nosocomial pathogen. Saudi Med J. 2023;44(9):825. doi:10.15537/smj.2023.44.9.20230216. 37717961 PMC10505283

[9] Gerding DN, Johnson S, Peterson LR, Mulligan ME, Silva J. Clostridium difficile-associated diarrhea and colitis. Infect Cont Hosp Ep. 1995;16(8):459–477. doi:10.2307/30141083. 7594392

[10] Lessa FC, Gould CV, McDonald LC. Current status of Clostridium difficile infection epidemiology. Clin Infect Dis. 2012;55(suppl_2):S65–S70. doi:10.1093/cid/cis319. 22752867 PMC3388017

[11] Maroo S, Lamont JT. Recurrent clostridium difficile. Gastroenterology. 2006;130(4):1311–1316. doi:10.1053/j.gastro.2006.02.044. 16618421

[12] Musher DM, Aslam S, Logan N, Nallacheru S, Bhaila I, Borchert F, Hamill RJ. Relatively poor outcome after treatment of Clostridium difficile colitis with metronidazole. Clin Infect Dis. 2005;40(11):1586–1590. doi:10.1086/430311. 15889354

[13] Walter J, Maldonado-Gómez MX, Martínez I. To engraft or not to engraft: an ecological framework for gut microbiome modulation with live microbes. Curr Opin Biotechnol. 2018;49:129–139. doi:10.1016/j.copbio.2017.08.008. 28866242 PMC5808858

[14] Erikstrup LT, Aarup M, Hagemann-Madsen R, Dagnaes-Hansen F, Kristensen B, Olsen KEP, Fuursted K. Treatment of Clostridium difficile infection in mice with vancomycin alone is as effective as treatment with vancomycin and metronidazole in combination. BMJ Open Gastroenterol. 2015;2(1):e000038. doi:10.1136/bmjgast-2015-000038. PMC4641438 26568840

[15] Cohen SH, Gerding DN, Johnson S, Kelly CP, Loo VG, McDonald LC, Pepin J, Wilcox MH. Clinical practice guidelines for Clostridium difficile infection in adults: 2010 update by the society for healthcare epidemiology of America (SHEA) and the infectious diseases society of America (IDSA). Infect Cont Hosp Ep. 2010;31(5):431–455. doi:10.1086/651706. 20307191

[16] Johnson S. 2016. Editorial commentary: potential risks and rewards with prophylaxis for Clostridium difficile infection. Clinical Infectious Diseases: Oxford University Press; p. 654–655. 10.1093/cid/ciw424 27358349

[17] Crawford T, Huesgen E, Danziger L. Fidaxomicin: a novel macrocyclic antibiotic for the treatment of Clostridium difficile infection. Am J Health Syst Pharm. 2012;69(11):933–943. doi:10.2146/ajhp110371. 22610025

[18] Mullane K. Fidaxomicin in Clostridium difficile infection: latest evidence and clinical guidance. Ther Adv Chronic Dis. 2014;5(2):69–84. doi:10.1177/2040622313511285. 24587892 PMC3926343

[19] Goldstein EJC, Babakhani F, Citron DM. Antimicrobial activities of fidaxomicin. Clin Infect Dis. 2012;55(suppl_2):S143–S148. doi:10.1093/cid/cis339. 22752863 PMC3388021

[20] Ajami NJ, Cope JL, Wong MC, Petrosino JF, Chesnel L. Impact of oral fidaxomicin administration on the intestinal microbiota and susceptibility to Clostridium difficile colonization in mice. Antimicrob Agents Chemother. 2018;62(5):e02112–17. doi:10.1128/AAC.02112-17. 29463537 PMC5923166

[21] Hurst A. 1981. Nisin. Advances in applied microbiology. Vol. 27, p. 85–123. Elsevier.

[22] Jančič U, Gorgieva S. Bromelain and nisin: the natural antimicrobials with high potential in biomedicine. Pharmaceutics. 2021;14(1):76. doi:10.3390/pharmaceutics14010076. 35056972 PMC8778819

[23] Shin JM, Gwak JW, Kamarajan P, Fenno JC, Rickard AH, Kapila YL. Biomedical applications of nisin. J Appl Microbiol. 2016;120(6):1449–1465. doi:10.1111/jam.13033. 26678028 PMC4866897

[24] Bartoloni A, Mantella A, Goldstein BP, Dei R, Benedetti M, Sbaragli S, Paradisi F. In-vitro activity of nisin against clinical isolates of Clostridium difficile. J Chemother. 2004;16(2):119–121. doi:10.1179/joc.2004.16.2.119. 15216943

[25] O’Reilly C, O’Connor PM, O’Sullivan Ó, Rea MC, Hill C, Ross RP. Impact of nisin on Clostridioides difficile and microbiota composition in a faecal fermentation model of the human colon. J Appl Microbiol. 2022b;132(2):1397–1408. doi:10.1111/jam.15250. 34370377

[26] van Kraaij C, Breukink E, Noordermeer MA, Demel RA, Siezen RJ, Kuipers OP, de Kruijff B. Pore formation by nisin involves translocation of its C-terminal part across the membrane. Biochemistry. 1998;37(46):16033–16040. doi:10.1021/bi980931b. 9819196

[27] Fooks LJ, Gibson GR. Mixed culture fermentation studies on the effects of synbiotics on the human intestinal pathogens Campylobacter jejuni and Escherichia coli. Anaerobe. 2003;9(5):231–242. doi:10.1016/S1075-9964(03)00043-X. 16887709

[28] Blanco-Míguez A, Beghini F, Cumbo F, McIver LJ, Thompson KN, Zolfo M, Manghi P, Dubois L, Huang KD, Thomas AM. Extending and improving metagenomic taxonomic profiling with uncharacterized species using MetaPhlAn 4. Nat Biotechnol. 2023;41(11):1633–1644. doi:10.1038/s41587-023-01688-w. 36823356 PMC10635831

[29] RStudio Team. *RStudio*: *integrated development for R*. *RStudio*; 2020.

[30] Wickham HG. 2016. 2: elegant graphics for data analysis. In: Data analysis. Springer International publishing; p. 189–201.

[31] Yadav MK, Tiwari SK. Methods for determination of antimicrobial activity of bacteriocins of lactic acid bacteria. Microbiology. 2023;92(6):745–765. doi:10.1134/S0026261723600520.

[32] Mathur H, Rea MC, Cotter PD, Hill C, Ross RP. The efficacy of thuricin CD, tigecycline, vancomycin, teicoplanin, rifampicin and nitazoxanide, independently and in paired combinations against Clostridium difficile biofilms and planktonic cells. Gut Pathog. 2016;8(1):1–10. doi:10.1186/s13099-016-0102-8. 27257437 PMC4890490

[33] Lay CL, Dridi L, Bergeron MG, Ouellette M, Fliss IL. Nisin is an effective inhibitor of Clostridium difficile vegetative cells and spore germination. J Med Microbiol. 2016;65(2):169–175. doi:10.1099/jmm.0.000202. 26555543

[34] Wood K. Microbial ecology: complex bacterial communities reduce selection for antibiotic resistance. Curr Biol. 2019;29(21):R1143–R1145. doi:10.1016/j.cub.2019.09.017. 31689403

[35] Mathur H, Mechoud MA, Matthews C, Lordan C, FitzGerald JA, Beresford T, Cotter PD. Methods to mitigate Escherichia coli blooms in human ex vivo colon model experiments using the high throughput micro-matrix bioreactor fermentation system. MethodsX. 2023;11:102393. doi:10.1016/j.mex.2023.102393. 37846356 PMC10577065

[36] O’Donnell MM, Rea MC, Shanahan F, Ross RP. The use of a mini-bioreactor fermentation system as a reproducible, high-throughput ex vivo batch model of the distal colon. Front Microbiol. 2018;9:391591. doi:10.3389/fmicb.2018.01844. PMC6096000 30147684

[37] Long B, Gottlieb M. Oral fidaxomicin versus vancomycin for Clostridioides difficile infection. Acad Emerg Med. 2022;29(12):1506–1507. doi:10.1111/acem.14600. 36156832

[38] Basolo A, Hohenadel M, Ang QY, Piaggi P, Heinitz S, Walter M, Walter P, Parrington S, Trinidad DD, von Schwartzenberg RJ. Effects of underfeeding and oral vancomycin on gut microbiome and nutrient absorption in humans. Nat Med. 2020;26(4):589–598. doi:10.1038/s41591-020-0801-z. 32235930

[39] Kim E, Kim AH, Lee Y, Ji SC, Cho J, Yu K, Chung J. Effects of vancomycin‐induced gut microbiome alteration on the pharmacodynamics of metformin in healthy male subjects. Clin Transl Sci. 2021;14(5):1955–1966. doi:10.1111/cts.13051. 33982376 PMC8504811

[40] Nazzal L, Soiefer L, Chang M, Tamizuddin F, Schatoff D, Cofer L, Aguero-Rosenfeld ME, Matalon A, Meijers B, Holzman R. Effect of vancomycin on the gut microbiome and plasma concentrations of gut-derived uremic solutes. Kidney Int Rep. 2021;6(8):2122–2133. doi:10.1016/j.ekir.2021.05.014. 34386661 PMC8343810

[41] Jarrad AM, Blaskovich MAT, Prasetyoputri A, Karoli T, Hansford KA, Cooper MA. Detection and investigation of eagle effect resistance to vancomycin in clostridium difficile with an ATP-bioluminescence assay. Front Microbiol. 2018;9:332066. doi:10.3389/fmicb.2018.01420. PMC6036128 30013531

[42] Marchandin H, Anjou C, Poulen G, Freeman J, Wilcox M, Jean-Pierre H, Barbut F. In vivo emergence of a still uncommon resistance to fidaxomicin in the urgent antimicrobial resistance threat Clostridioides difficile. J Antimicrob Chemother. 2023;78(8):1992–1999. doi:10.1093/jac/dkad194. 37352110

[43] Cao X, Boyaci H, Chen J, Bao Y, Landick R, Campbell EA. Basis of narrow-spectrum activity of fidaxomicin on Clostridioides difficile. Nature. 2022;604(7906):541–545. doi:10.1038/s41586-022-04545-z. 35388215 PMC9635844

[44] Costa DVS, Pham NVS, Hays RA, Bolick DT, Goldbeck SM, Poulter MD, Hoang SC, Shin JH, Wu M, Warren CA. Influence of binary toxin gene detection and decreased susceptibility to antibiotics among Clostridioides difficile strains on disease severity: a single-center study. Antimicrob Agents Chemother. 2022;66(8):e00489–22. doi:10.1128/aac.00489-22. 35861541 PMC9380565

[45] Zhanel GG, Walkty AJ, Karlowsky JA. Fidaxomicin: a novel agent for the treatment of Clostridium difficile infection. Can J Infect Dis Med Microbiol. 2015;26(6):305–312. doi:10.1155/2015/934594. 26744587 PMC4692299

[46] Baines SD, Crowther GS, Freeman J, Todhunter S, Vickers R, Wilcox MH. SMT19969 as a treatment for Clostridium difficile infection: an assessment of antimicrobial activity using conventional susceptibility testing and an in vitro gut model. J Antimicrob Chemother. 2015;70(1):182–189. doi:10.1093/jac/dku324. 25190720 PMC4267497

[47] Weiss W, Pulse M, Vickers R. In vivo assessment of SMT19969 in a hamster model of Clostridium difficile infection. Antimicrob Agents Chemother. 2014;58(10):5714–5718. doi:10.1128/AAC.02903-14. 25022586 PMC4187990

[48] Collins DA, Riley TV. Ridinilazole: a novel, narrow‐spectrum antimicrobial agent targeting Clostridium (Clostridioides) difficile. Lett Appl Microbiol. 2022;75(3):526–536. doi:10.1111/lam.13664. 35119124 PMC9541751

[49] Goldstein EJC, Citron DM, Tyrrell KL, Merriam CV. Comparative in vitro activities of SMT19969, a new antimicrobial agent, against Clostridium difficile and 350 gram-positive and gram-negative aerobic and anaerobic intestinal flora isolates. Antimicrob Agents Chemother. 2013;57(10):4872–4876. doi:10.1128/AAC.01136-13. 23877700 PMC3811411

[50] Bublitz A, Brauer M, Wagner S, Hofer W, Müsken M, Deschner F, Lesker TR, Neumann-Schaal M, Paul L-S, Nübel U. et al. The natural product chlorotonil a preserves colonization resistance and prevents relapsing Clostridioides difficile infection. Cell Host & Microbe. 2023;31(5):734–750.e8. doi:10.1016/j.chom.2023.04.003. 37098342

[51] Gerth K, Steinmetz H, Höfle G, Jansen R. Chlorotonil A, a macrolide with a unique gem‐dichloro‐1, 3‐dione functionality from sorangium cellulosum, so ce1525. Angew Chem Int Ed. 2008;47(3):600–602. doi:10.1002/anie.200703993. 18058875

[52] Jungmann K, Jansen R, Gerth K, Huch V, Krug D, Fenical W, Müller R. Two of a kind the biosynthetic pathways of chlorotonil and anthracimycin. ACS Chem Biol. 2015;10(11):2480–2490. doi:10.1021/acschembio.5b00523. 26348978

